# Updates on the role of adrenal steroidogenesis inhibitors in Cushing’s syndrome: a focus on novel therapies

**DOI:** 10.1007/s11102-016-0742-1

**Published:** 2016-09-06

**Authors:** Maria Fleseriu, Frederic Castinetti

**Affiliations:** 1 Departments of Medicine and Neurological Surgery, and Northwest Pituitary Center, Oregon Health & Science University, Mail Code: CH8N, 3303 SW Bond Ave, Portland, OR 97239 USA; 2 Aix Marseille University, CNRS, CRN2M, Department of Endocrinology, Assistance Publique Hopitaux de Marseille, Marseille, France

**Keywords:** Cushing’s disease, Cushing’s syndrome, Adrenal steroidogenesis inhibitor, Osilodrostat, Levoketoconazole, LCI699, Ketoconazole, Metyrapone

## Abstract

**Purpose:**

Endogenous Cushing’s syndrome (CS) is a rare disease that results from exposure to high levels of cortisol; Cushing’s disease (CD) is the most frequent form of CS. Patients with CS suffer from a variety of comorbidities that increase the risk of mortality. Surgical resection of the disease-causing lesion is generally the first-line treatment of CS. However, some patients may not be eligible for surgery due to comorbidities, and approximately 25 % of patients, especially those with CD, have recurrent disease. For these patients, adrenal steroidogenesis inhibitors may control cortisol elevation and subsequent symptomatology. CS is rare overall, and clinical studies of adrenal steroidogenesis inhibitors are often small and, in many cases, data are limited regarding the efficacy and safety of these treatments. Our aim was to better characterize the profiles of efficacy and safety of currently available adrenal steroidogenesis inhibitors, including drugs currently in development.

**Methods:**

We performed a systematic review of the literature regarding adrenal steroidogenesis inhibitors, focusing on novel drugs.

**Results:**

Currently available adrenal steroidogenesis inhibitors, including ketoconazole, metyrapone, etomidate, and mitotane, have variable efficacy and significant side effects, and none are approved by the US Food and Drug Administration for CS. Therefore, there is a clear need for novel, prospectively studied agents that have greater efficacy and a low rate of adverse side effects. Efficacy and safety data of current and emerging adrenal steroidogenesis inhibitors, including osilodrostat (LCI699) and levoketoconazole (COR-003), show promising results that will have to be confirmed in larger-scale phase 3 studies (currently ongoing).

**Conclusions:**

The management of CS, and particularly CD, remains challenging. Adrenal steroidogenesis inhibitors can be of major interest to control the hypercortisolism at any time point, either before or after surgery, as discussed in this review.

## Introduction

Cushing’s syndrome (CS) is a potentially fatal disease associated with chronic hypercortisolemia [1]. Endogenous CS is rare, with an annual estimated incidence of approximately 0.7–2.4 cases per million people [1]. Patients with CS have a mortality risk that is approximately 3.5–5 times higher than the general population if not appropriately treated [2]. The increased mortality with CS is, in part, related to an increased risk of cardiovascular disease and coagulation disorders [3–5]. The risk of myocardial infarction is approximately 4.5 times higher, and the risk of venous thromboembolism is approximately 20.6 times higher in patients with CS compared with the general population [2]. Elevated cortisol levels in patients with CS also result in a range of comorbidities, including cardiovascular (e.g., venous thrombosis, hypertension), dermatological (e.g., plethora, ecchymoses), metabolic (e.g., weight gain, abnormal glucose tolerance), reproductive (e.g., decreased libido, menstrual changes), neuropsychiatric (e.g., lethargy, depression), and musculoskeletal (e.g., muscle weakness) symptoms [1].

Treatment options that directly address the hypercortisolemia include surgery (pituitary, adrenal, or ectopic tumor resection), medical treatment, and/or radiotherapy [6, 7]. Each option has its drawbacks; for instance, radiation techniques become fully effective only after a prolonged period of time (3–5 years) and medical treatment, as a bridge is necessary. Focusing more specifically on Cushing’s disease (CD), the most frequent etiology of CS, the 2015 Endocrine Society Clinical Practice Guideline recommends surgical resection of the pituitary lesion as a first line of treatment, unless surgery is contraindicated or unlikely to successfully reduce excess cortisol levels [6]. For patients for whom disease was not controlled by initial surgery, or for patients with severe, life-threatening disease, bilateral adrenalectomy is also an option [6]. However, as a result of the adrenal insufficiency (AI) induced by bilateral adrenalectomy, patients undergoing the procedure will require hydrocortisone replacement and have an increased risk of adrenal crises during their lifetime [8].

Medical treatment is recommended in patients who are not surgical candidates, or for whom surgery has failed, and in patients awaiting the effects of radiation therapy/radiosurgery [6].

There are three specific targets for medical therapy in CD, the corticotroph tumor, adrenal steroidogenesis inhibitors, and glucocorticoid receptor blockers.

Steroidogenesis inhibitors are recommended by the Endocrine Society as second-line treatment after adenomectomy in CD, depending on clinical circumstances; as first-line treatment for patients with ectopic adrenocorticotropic hormone (ACTH) secretion when a tumor is not detected; or as an adjunct treatment for patients with adrenocortical carcinoma [6].

**Fig. 1:**
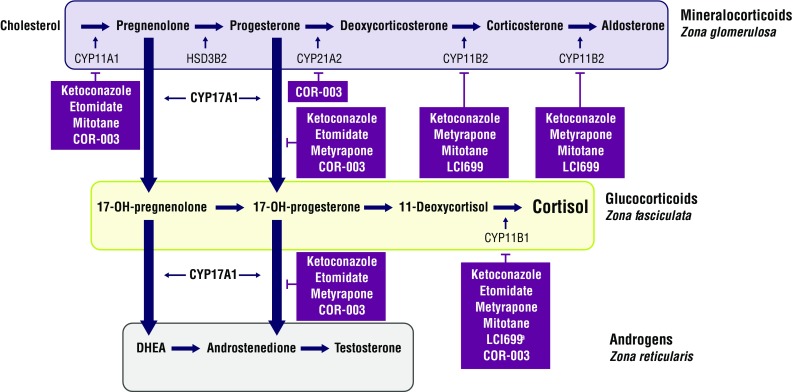
Effects of adrenal steroidogenesis inhibitors on the cortisol synthesis pathway [9]. The enzymatic pathways in cortisol synthesis are represented schematically. Inhibitors are depicted in *purple boxes* and may inhibit multiple steps in the pathway. ^a^At high doses. CYP, cytochrome P450

Corticotropin (ACTH) stimulates steroidogenesis by the adrenal glands [9]. Through a variety of enzymatic reactions, cholesterol, the common steroid precursor, is converted to aldosterone, cortisol, or androstenedione (Fig. 1). Adrenal steroidogenesis inhibitors, which act by blocking various steps in the steroid biosynthesis pathway resulting in reduced production of cortisol and other steroids, are a cornerstone of medical treatment of CS [10]. This review summarizes the key features of different adrenal steroidogenesis inhibitors for the treatment of CS, with particular emphasis on steroidogenesis inhibitors currently in clinical development. Mechanisms of action, efficacy, and safety of the adrenal steroidogenesis inhibitors are discussed in detail below and summarized in Table 1.

**Table 1:** Mechanisms of action of steroidogenesis inhibitors [21]

	Mechanism of action	Efficacy (%)	Common side effects
Ketoconazole	Inhibitor of CYP17A1, CYP11A1, and CYP11B1	53–88	Liver enzyme increase
			Gastrointestinal AEs
			Interactions with multiple drugs
			Not approved for use during pregnancy
Metyrapone	Inhibitor of CYP11B1, CYP11B2, and CYP17A1	75	Hypokalemia
			Hypertension
			Gastrointestinal AEs
			Hirsutism
			Not approved for use during pregnancy
Etomidate	Inhibitor of CYP11B1, CYP17A1, and CYP11A1	NR	Hypnosis
			Not approved for use during pregnancy
Mitotane	Inhibitor of CYP11A1, CYP11B1, and CYP11B2	~70	Gastrointestinal AEs
			Neurological side effects
			Teratogen (not approved for use during pregnancy)
Osilodrostat (LCI699)	Inhibitor of CYP11B2 and CYP11B1 at higher doses	78–92	Nausea
			Hirsutism
			Fatigue
			Headache
			Hypokalemia
			Not approved for use during pregnancy
Levoketoconazole (COR-003)	Inhibitor of CYP17A1, CYP11A1, CYP11B1, and CYP21A2	NR	Headache^a^
			Nausea^a^
			Mild liver enzyme increase^a^
			Not approved for use during pregnancy

CYP, cytochrome P450; AE, adverse event; NR, not reported. All of these drugs can induce adrenal insufficiency

^a^In patients with diabetes mellitus

## Adrenal steroidogenesis inhibitors currently in clinical use

### Ketoconazole

Ketoconazole, a synthetic imidazole derivative, is an antifungal that, at higher doses (400–1200 mg), reduces adrenal steroid production [11]. However, due to liver toxicity, the approved use of ketoconazole in the United States (US) is restricted to the treatment of serious fungal infections with no other viable treatment options [7, 12]. Ketoconazole is approved for the treatment of CS in the European Union [13]. While not approved for the treatment of CS by the US Food and Drug Administration (FDA), ketoconazole is one of the most commonly used steroidogenesis inhibitors for off-label treatment of CS [11].

Ketoconazole inhibits key cytochrome P450 (CYP) enzymes involved in multiple steps of steroidogenesis in the adrenal cortex, including CYP17A1, CYP11A1, CYP11B1, and CYP11B2 [10, 14, 15]. Ketoconazole is a 50/50 racemic mixture of 2S,4R and 2R,4S enantiomers [16], and these enantiomers exhibit differences in inhibitory potency for the enzymes involved in steroidogenesis [17–19]. Ketoconazole has also been reported to directly inhibit ACTH secretion [20], although these findings have not been confirmed [21].

In patients with CS, ketoconazole treatment has been associated with significant decreases in urinary free cortisol (UFC) and urinary levels of cortisol and androgen metabolites [22]. A retrospective study of 200 patients receiving single-agent ketoconazole reported that 50 % of the patients had normal UFC at the end of the study, while 26 % of “uncontrolled” patients had a ≥50 % decrease in UFC; concurrent improvements in hypertension, diabetes, and hypokalemia were also observed [23]. Escape from ketoconazole-mediated control occurred in some patients (7 %) [8]. However, interestingly, 50 % of the patients treated for more than 24 months (mean = 108 months) remained controlled with a stable dose of ketoconazole [23].

Elevations in liver enzymes occurred in approximately 15 % of patients treated with ketoconazole [23, 24]. Liver enzyme levels returned to normal within 1–4 weeks after lowering the dose or discontinuing treatment, and severe drug-induced liver injury was rare [25]. In this retrospective analysis, no fatal hepatitis was observed; however, a dramatic (~40-fold) increase in liver enzymes was observed in one patient who was consuming concomitant alcohol and high-dose acetaminophen [23]. Similarly, in retrospective chart review, elevation of liver enzymes and severe acute liver injury were rarely observed with ketoconazole treatment [26].

Concomitant use of drugs with known hepatotoxic effects should be avoided, and acid-lowering drugs should be used with caution, as they decrease the efficacy of ketoconazole [24]. Adrenal insufficiency is rare, except when the treatment is given as a block-and-replace strategy (a regimen in which cortisol production is completely inhibited by steroidogenesis inhibitors and glucocorticoids are administered to fulfill physiologic needs) [23]. Ketoconazole may also affect gonadal testosterone synthesis, resulting in decreased androgen levels and subsequent hypogonadism and gynecomastia in male patients; therefore, it is generally used as a second-line medical therapy in men (patients in whom metyrapone might be more appropriate, from this point of view) [11, 25].

### Metyrapone

Metyrapone is FDA approved for the diagnosis of AI in the US [27] and is used clinically, off label, for the treatment of CS [10]. In the European Union, metyrapone is approved for the treatment of CS [13]. Metyrapone exhibits potent, relatively selective inhibition of CYP11B1, but also inhibits the activity of CYP11B2 [28]. Significant reductions in urinary secretion of cortisol and aldosterone have been observed with metyrapone treatment [29]. In a retrospective study of 164 patients with CS who received metyrapone monotherapy, 43–76 % of patients achieved control of cortisol levels (defined by prespecified values of mean serum cortisol day-curve, 24-hour UFC, and 09.00 h cortisol) [30]. No escape was reported, and 83 % of the patients treated for more than 12 months (mean = 22 months) were controlled on a stable dose of metyrapone [30].

Metyrapone has been associated with gastrointestinal adverse events and hypoadrenalism [6, 30]. Despite the accumulation of adrenogenic and mineralocorticoid precursors associated with metyrapone treatment, incidence of metyrapone-related hirsutism, acne, and edema were rare, albeit not prospectively studied, and hypokalemia was reported but manageable with replacement [30]. Of note, some patients were initially treated with antialdosterone drugs, which might have led to an underestimation of worsening of hypokalemia and hypertension. Although no clear recommendation has been made on this specific point, metyrapone is probably a better choice for a second-line medical treatment in females for whom a long-term treatment is necessary because of hyperandrogenism [6].

### Etomidate

Etomidate, an imidazole derivative, is used for the induction of anesthesia and is also a potent and dose-dependent inhibitor of CYP11B1, CYP17A1, and CYP11A1 [6, 21, 31, 32]. Etomidate can be administered intravenously and is often used for seriously ill patients with severe hypercortisolemia who cannot take oral medication [6, 21]. However, only a few cases of its use have been reported in the literature.

Earlier studies demonstrated that inhibition of cortisol was rapidly achieved with low-dose (2.5 mg/h) etomidate in patients with hypercortisolism and was distinct from the sedative effects of the drug [33]. In patients with CS, etomidate treatment resulted in significant suppression of serum cortisol levels within 11 h of infusion [34]. In an emergency setting, patients with CS who received etomidate at a dose of 0.1 mg/kg per hour exhibited rapid and prolonged suppression of serum cortisol levels [34]. The most common side effects associated with etomidate were hypnotic effect, reduced blood pressure, myoclonus, dystonia, nausea, and vomiting [31, 33]. Adrenal insufficiency has also been reported, which may require glucocorticoid replacement [31], thus a block-and-replace protocol is used in most cases. Etomidate is unstable in water, and is often administered in a formulation containing propylene glycol, which may increase the incidence of hemolysis and nephrotoxicity [33].

### Mitotane

Mitotane, a synthetic derivative of the pesticide dichlorodiphenyltrichloroethane, is indicated for the treatment of adrenocortical carcinoma, but in rare cases may be used for the treatment of hypercortisolemia [10, 35]. Mitotane inhibits CYP11A1, CYP11B1, CYP11B2, and 5α-reductase [7, 10]. In patients with CS, mitotane treatment has been associated with significant reductions in cortisol and androgen levels [36]. In the largest study (76 patients) reported to date on the use of mitotane in CD, control of cortisol hypersecretion was observed in 72 % of the patients after a median time of 6.7 months; the mitotane level necessary to obtain control was lower than the level recommended for the treatment of adrenal carcinoma (8.5 vs 14 µg/L, respectively). Interestingly, 75 % of the patients needed to be treated with hydrocortisone in parallel, probably due to adrenal atrophy, but this effect was transient in the majority of the patients, as withdrawal of the drug led to recurrence in 71 % of them [37]. Results were similar in patients treated for ectopic ACTH secretion; 21/23 patients (91 %) achieved normal UFC levels after a mean time of 4.5 months [38].

Mitotane is associated with a number of potential side effects, including hypercholesterolemia, anorexia, gastrointestinal symptoms, decreased memory and other neurological side effects, and abnormal liver function [31, 38]; these side effects lead to discontinuation of treatment in approximately a quarter of patients and require close monitoring of plasma mitotane levels [6]. A recent study of premenopausal women demonstrated a high incidence of menstrual disorders and ovarian macrocysts in women receiving mitotane, which may be related to elevated levels of luteinizing hormone, follicle-stimulating hormone, and estrogen as a consequence of mitotane alleviating the negative feedback normally exerted by the ovaries on the production of those hormones [39].

## Adrenal steroidogenesis inhibitors in clinical development

### Osilodrostat (LCI699)

Osilodrostat is a potent and relatively selective inhibitor of CYP11B2 that also inhibits CYP11B1 at higher doses [40]. In comparison to metyrapone, which also inhibits CYP11B1, osilodrostat is a more potent inhibitor of CYP11B1 (IC_50_ 2.5 vs 7.5 nM) and has a longer half-life (4–5 vs 2 h) [41]. Osilodrostat was initially developed as a possible treatment option for hypertension, cardiac failure, and renal disease [10]. In studies in patients with hypertension, significant and dose-dependent decreases in urine and plasma levels of aldosterone and a blunting of the cortisol response to synthetic ACTH were observed [10].

In a 10-week, proof-of-concept study in patients with CD (n = 12) who had previously undergone pituitary surgery and had UFC greater than 1.5 times the upper limit of normal (ULN), 92 % of patients achieved normalization of UFC within 10 weeks of initiating osilodrostat treatment, with all patients achieving ≥50 % decreases in UFC levels from baseline [42]. After treatment discontinuation, UFC levels rose above the ULN [42]. In a longer term, 22-week, phase 2 study of osilodrostat in patients with CD with UFC levels above the ULN, normalization of cortisol levels was achieved in 84 % of patients by week 10 and 79 % by the end of the study [41]. In both studies, patients achieved normal UFC within a month of starting treatment [41, 42]. Plasma levels of cortisol and aldosterone were decreased in both studies, while levels of their precursors, 11-deoxycortisol and 11-deoxycorticosterone, increased [41, 42]. Although blood pressure decreased from baseline in the proof-of-concept study, data from the phase 2 study showed no changes in blood pressure [41, 42].

In patients with CD, osilodrostat is also associated with an increase in ACTH (3- to 4-fold), which, due to the subsequent increase in 11-deoxycorticosterone, may be associated with the development of certain side effects (worsening of hypokalemia, increased blood pressure levels) [10]. These side effects were not specifically reported in the studies previously described. Adrenal insufficiency was observed in approximately 32 % of patients in the phase 2 study [41]. The most common adverse events in both studies were gastrointestinal adverse events (e.g., nausea, diarrhea); adverse events associated with adrenal insufficiency (i.e., fatigue, dizziness, nausea, muscle spasms, hypotension, and syncope) led to dose reductions of osilodrostat [41, 42]. In both studies, increased levels of testosterone were observed in female patients [41, 42], and in the phase 2 study, symptoms of hirsutism occurred in a third of the female patients who completed the study [41].

A phase 3, double-blind, randomized withdrawal study (ClinicalTrials.gov identifier NCT02180217) of osilodrostat in patients with persistent, recurrent CD or untreated CD who are not candidates for surgery is ongoing (estimated enrollment = 132) [43]. Patients receive osilodrostat during a 12-week, open-label, dose-titration period (weeks 1–12), followed by a 12-week, open-label, stable treatment period (weeks 13–24); then patients are randomized to receive osilodrostat or a placebo during an 8-week double-blind period (weeks 26–34), and, finally, all patients receive open-label osilodrostat from weeks 35 to 48. The primary endpoint is the proportion of patients with normal mean UFC values per treatment group at 34 weeks.

### Levoketoconazole (COR-003)

Levoketoconazole is the 2S,4R enantiomer of ketoconazole, purified from racemic ketoconazole [44]. In early in vitro analyses, levoketoconazole was shown to be a more potent inhibitor than the 2R,4S enantiomer of several enzymes in the steroidogenic pathway, including CYP11B1, a key enzyme in the final step of adrenal cortisol synthesis; levoketoconazole had a half maximal inhibitory concentration for CYP11B1 that was approximately 4 times lower than the 2R,4S enantiomer [17–19]. As an azole antifungal, levoketoconazole was screened as an inhibitor of a critical enzyme for fungal survival, CYP51A1, which also happens to be a key enzyme in cholesterol synthesis. Levoketoconazole was shown to be a more potent inhibitor of CYP51A1 than the 2R,4S enantiomer [19]. Results of more recent analyses of the effects of racemic ketoconazole, levoketoconazole, and 2R,4S-ketoconazole on steroidogenic CYP enzymes support those of early in vitro studies [45]. In those analyses, levoketoconazole showed more potent inhibition of CYP17A1, CYP21A2, and CYP11B1 than either racemic ketoconazole or 2R,4S-ketoconazole [45]. Thus, a lower dose of levoketoconazole may potentially be used to achieve the same clinical effects as racemic ketoconazole; however, controlled clinical trials of different doses of levoketoconazole compared with racemic ketoconazole are needed to support these findings. In preclinical studies in rats, levoketoconazole had a greater potency for decreasing corticosterone (the main glucocorticoid in rats [46]) and testosterone levels than the 2R,4S enantiomer or racemic ketoconazole [45]. Levoketoconazole-mediated decreases in corticosterone were dose-dependent. Levoketoconazole treatment has not yet been evaluated in patients with CS; however, clinical results in healthy subjects and patients with type 2 diabetes support preclinical results showing suppression of corticosterone by levoketoconazole. In an analysis of cortisol levels over time in healthy subjects, the mean area under the curve (AUC) for cortisol was lower following administration of both levoketoconazole and ketoconazole compared with placebo. In a study in patients with type 2 diabetes, nonsignificant mean reductions in 12-hour overnight plasma cortisol AUC were observed with both ketoconazole and levoketoconazole after 14 days of dosing [47]. Interestingly, no dose-dependent trend in the percentage change in testosterone was found with levoketoconazole administration [47]. Finally, significant decreases in low-density lipoprotein cholesterol levels were observed in diabetic patients treated with levoketoconazole [47]. Levoketoconazole treatment also resulted in nonsignificant lower serum cholesterol levels in rats than the 2R,4S enantiomer or racemic ketoconazole. This may be of particular clinical relevance for patients with CS who have a substantially increased risk of cardiovascular events [2].

In patients with type 2 diabetes, headache and nausea were the most commonly reported adverse events [47]. Although levoketoconazole treatment has not yet been evaluated in patients with CS, levoketoconazole may have a more favorable safety profile than racemic ketoconazole, which has been associated with clinically relevant drug–drug interactions and hepatotoxicity. The pharmacokinetic profiles of levoketoconazole and the 2R,4S enantiomer suggest preferred extraction of the 2R,4S enantiomer by the liver, which may indicate a lower risk of hepatotoxicity with levoketoconazole [45]. In addition, levoketoconazole shows less potent inhibition of CYP7A1, a rate-limiting enzyme in bile acid synthesis, than the 2R,4S enantiomer [47]. Nevertheless, there has been some clinical evidence of alterations in liver function enzymes with levoketoconazole treatment [47, 48]; further studies will be needed to clarify whether decreased hepatotoxicity is observed with levoketoconazole in patients with CS. A single-arm, open-label, dose-titration study evaluating levoketoconazole in patients with persistent or recurrent CS, or newly diagnosed patients who are not candidates for surgery, is ongoing (ClinicalTrials.gov identifier NCT01838551) [44]. The study has a variable dose-titration phase, a 6-month maintenance phase in which patients receive levoketoconazole at the therapeutic dose, and a 6-month extended evaluation phase. The primary endpoint is the rate of normalized 24-hour UFC at 6 months of maintenance without dose increase; safety issues are closely monitored in this study.

## Clinical considerations for treatment with adrenal steroidogenesis inhibitors in CS

### Drug interactions

Most adrenal steroidogenesis inhibitors have the potential for drug interactions with drugs metabolized by CYP enzymes (Table 2). Both mitotane and ketoconazole are strong inhibitors of CYP3A4 and may enhance the activity of drugs metabolized by CYP3A4, including oral anticoagulants, statins, cyclosporine, and tacrolimus [49–51]. The inhibitory effects of mitotane treatment on CYP3A4 are long lasting and have been observed several months after therapy is discontinued [51]. Levoketoconazole has similar effects to those of racemic ketoconazole on the CYP enzymes most relevant to drug metabolism [45]. Like ketoconazole, levoketoconazole has the potential for drug interactions with drugs metabolized via CYP3A4 (e.g., felodipine and atorvastatin) [45]. In drug interaction studies, exposure of felodipine was increased 10-fold with coadministration of levoketoconazole, while exposure to atorvastatin was increased by ~30 % when coadministered with levoketoconazole (compared with a ~50 % increase with coadministration of racemic ketoconazole). In vitro data have also indicated that ketoconazole inhibits the cardiac potassium channel, hERG, which may result in prolongation of QT interval; thus, ketoconazole should be used with caution when used in combination with agents that also prolong QT interval or are metabolized by CYP3A4 [52]. In studies of healthy subjects, ketoconazole significantly increased QT interval [53, 54]. A trend toward prolongation of QT interval was also reported in a study of levoketoconazole in healthy subjects [45]. While it is likely that the effect of levoketoconazole on QT interval will be similar to that of ketoconazole, additional studies are needed. Mifepristone, a glucocorticoid receptor antagonist, is also metabolized by CYP3A; thus, drugs that inhibit CYP3A, such as ketoconazole and levoketoconazole, may increase plasma mifepristone concentrations [55]. Coadministration of these drugs is rare; however, it is important to ensure an adequate wash-out when switching from one therapy to another.

**Table 2:** Drug–drug interactions with adrenal steroidogenesis inhibitors

Medical intervention	Interacts with	Potential for adverse event
Ketoconazole [24, 54, 69]	CYP3A4	Drug–drug interactions with oral anticoagulants, statins, cyclosporine, and tacrolimus
	hERG	Possible QT prolongation
		Use acid-lowering and hepatotoxic drugs with caution
Metyrapone [68]	UGT1	Drug–drug interactions occur frequently
		Acetaminophen toxicity
Etomidate [70–73]	CYP11B1	Etomidate should be given carefully with calcium channel blockers, opioids, and benzodiazepines
Mitotane [35]	CYP3A4	Drug–drug interactions with oral anticoagulants, statins, cyclosporine, and tacrolimus
		Hydrocortisone dose increased
Osilodrostat (LCI699) [21, 60, 74]	CYP1A2 and CYP2C19 (moderate inhibition) CYP2D6 and CYP3A4 (weakly inhibited)	Drug–drug interactions Possible QT prolongation
Levoketoconazole (COR-003) [21]	CYP3A4, CYP3A5 hERG	Drug–drug interactions with oral anticoagulants, statins, cyclosporine, and tacrolimus
		QT prolongation

CYP, cytochrome P450; hERG, human ether-a-go–go–related gene; UGT1, UDP-glucuronosyltransferase 1 family; NR, not reported

### Combination therapy

Steroidogenic inhibitors are typically administered as monotherapy [6]. Combination therapy may be necessary in patients who do not respond to monotherapy or who experience dose-limiting side effects [10]. In patients with severe CS, combining more than two adrenal steroidogenesis inhibitors has also been shown to be effective. Combination therapy with ketoconazole and cabergoline normalized UFC in 79 % of patients with CD; however, based on late-night salivary cortisol levels, subclinical hypercortisolism persisted in patients treated with this combination [56]. In a study of 17 patients with CD, treatment with a regimen of pasireotide, cabergoline, and ketoconazole normalized UFC levels in 88 % of patients by day 80; reductions in UFC levels of up to 67 % were observed in patients with mild (1 to <2 times ULN) or severe (4–6 times ULN) CD [57]. Combination therapy with ketoconazole and metyrapone is relatively common, but is generally only considered for patients with more severe disease or for those who have failed to respond to monotherapy with either agent [10, 30]. Due to the relatively slow onset of action of mitotane, combination therapy with a rapid-onset steroidogenesis inhibitor may be used during the first several months of treatment [10]. In patients with severe hypercortisolism (UFC >5 times ULN) related to ectopic ACTH syndrome or adrenal carcinoma, the combination of metyrapone and ketoconazole (and in some patients, subsequent mitotane) resulted in normal UFC values in 73 % of patients (n = 22) and a concomitant improvement of clinical symptoms, including hypokalemia, hypertension, and diabetes [58]. Similarly, in a study of 11 patients with severe hypercortisolism and significant comorbidities that precluded surgical intervention, who were administered metyrapone and ketoconazole followed by mitotane maintenance therapy, UFC was normalized in all patients receiving combination therapy and remained below the ULN on maintenance therapy; all patients were able to undergo surgery 5–22 months after beginning combination therapy [59].

In mice, the combination of osilodrostat and pasireotide was similar to the toxicity of either agent alone and appeared to ameliorate the cellular hypertrophy associated with osilodrostat therapy and the decrease in liver weight following pasireotide therapy [60]. Additional studies are needed in patients with CD to confirm these results. Combination therapy with levoketoconazole has not yet been evaluated in patients with CS; however, based on the in vitro data, it is hypothesized that the greater potency of levoketoconazole for cortisol suppression relative to ketoconazole, along with its potentially favorable hepatic tolerability profile, may make levoketoconazole a better option for combination therapy.

### Preoperative treatment

The goal of presurgical medical treatment is to decrease the comorbidities at the time of surgery and improve postoperative outcomes. Preoperative treatment could thus be of particular interest in patients with comorbidities that are difficult to manage with classical antihypertensive or antidiabetic treatments. Results of preoperative therapy with adrenal steroidogenesis inhibitors have not been conclusive because some patients were only evaluated based upon the rate of normal UFC obtained before surgery and not on the rate of improvement of comorbidities [61]. In a retrospective study of 16 patients who received adequate presurgical cortisol suppression therapy with ketoconazole or metyrapone prior to undergoing transsphenoidal surgery, postoperative cortisol suppression was observed and, surprisingly, long-term remission was significantly increased compared with patients with borderline or inadequate cortisol suppression pretreatment [62]. However, a retrospective study of previously untreated patients who had a mean baseline UFC level of 793 nmol/24 h (approximately 3 times ULN) and who were treated with steroidogenic inhibitors prior to surgery found that preoperative treatment with ketoconazole and/or metyrapone yielded normal UFC levels in approximately 50 % of patients, but normalization of UFC did not necessarily lead to clinical benefit [61]. Further studies with more well-defined patient inclusion criteria will be required to clarify the role of presurgical treatment. Moreover, the duration of this presurgical treatment is also controversial. Taking into account the time needed to recover from comorbidities, for instance after bilateral adrenalectomy, would argue for the need for a prolonged presurgical medical treatment [63].

### Subclinical adrenal CS

Though controversial, subclinical CS or mild adrenal hypercortisolism is defined as a subtle hypersecretion of cortisol that does not fully manifest in a clinical phenotype [64]. However, subclinical CS may be associated with an increased risk of comorbidities, such as hypertension, type 2 diabetes, osteoporosis, dyslipidemia, or coronary heart disease as a result of chronic cortisol elevation, but this association has not been conclusively demonstrated. As a result of the sparse data around treatment of subclinical CS, treatment guidelines for subclinical CS vary [65]. Some guidelines recommend adrenalectomy, particularly for patients with worsening comorbidities, while others advocate for careful monitoring of the patient [6, 66, 67]. While the optimal treatment for these patients is not clear, steroidogenic inhibitors are not currently recommended for the treatment of subclinical CS. Thus, there is a clear need for more studies evaluating the best course of treatment for these patients. For instance, administering steroidogenesis inhibitors as a first-line treatment might obviate the need for adrenalectomy in patients who have bilateral adrenal disease, have contraindications, or refuse surgery, if their comorbidities are improved with the medical treatment.

## Conclusions

CS has higher morbidity and mortality rates if not appropriately treated. Surgery is the first line of treatment, but many patients do not achieve remission or experience disease recurrence following surgery. Adrenal steroidogenesis inhibitors are associated with high rates of cortisol normalization and clinical improvements. Combination therapies of well-studied drugs might allow for lower doses with better tolerability. However, data from CS studies is limited and there is a need for prospective studies of adrenal steroidogenesis inhibitors with long-term follow-up. In addition, agents with improved tolerability and potency are needed. In phase 2 studies, osilodrostat provided cortisol normalization in almost 80 % of patients with no short-term escape. Based on preclinical and early clinical data in patients with diabetes, levoketoconazole may be associated with a number of potential benefits relative to steroidogenesis inhibitors currently in clinical use. Both drugs are currently being evaluated in ongoing phase 3 studies. Treatments that are tailored to each individual patient’s needs are desirable; thus, expanded options for medical treatment could lead to improved overall outcomes for CS patients.
